# Supplementary material for: Cardiac and Vascular α1-Adrenoceptors in Congestive Heart Failure: A Systematic Review
Source: Cells. 2020 Nov 4;9(11):2412. doi: 10.3390/cells9112412 (PMC7694190; doi:10.3390/cells9112412)
Supplement: Supplementary file 1 [file cells-09-02412-s001.pdf]

cells

Review

Cardiac and Vascular α_1-Adrenoceptors in Congestive Heart Failure: A Systematic Review

Gizem Kaykı-Mutlu [1,†], Olga Papazisi [2,†], Meindert Palmen [2], A.H. Jan Danser [3], Martin C. Michel [4,*] and Ebru Arioglu-Inan [1]

1 Department of Pharmacology, Faculty of Pharmacy, Ankara University, 06560, Ankara, Turkey; gkayki@ankara.edu.tr (G.K.M.); ebru.arioglu@ankara.edu.tr (E.A.I.)
2 Department of Cardiothoracic Surgery, Leiden University Medical Center; 2300 RC, Leiden, The Netherlands, o.papazisi@lumc.nl (O.P.); M.Palmen@lumc.nl (M.P.)
3 Department of Internal Medicine, Division of Pharmacology, Erasmus Medical Center, 3000 CA, Rotterdam,The Netherlands, a.danser@erasmusmc.nl
4 Department of Pharmacology, Johannes Gutenberg University, 55131, Mainz, Germany
* Correspondence: marmiche@uni-mainz.de
† These authors have contributed equally.

Appendix A

Full Search Strategy

((("Heart Failure"[mesh] OR "heart failure"[tw] OR "cardiac failure"[tw] OR "myocardial failure"[tw] OR "heart decompensation"[tw] OR "heart decompensation"[tw] OR "cardio-renal syndrome"[tw] OR "cardiorenal syndrome"[tw] OR "paroxysmal dyspnea"[tw] OR "cardiac edema"[tw] OR "cardiac oedema"[tw] OR "heart edema"[tw] OR "heart oedema"[tw] OR "failing myocardium"[tw] OR "failing heart"[tw] OR "failing human myocardium"[tw] OR "failing human heart"[tw]) AND ("Receptors, Adrenergic, alpha 1"[Mesh] OR "Adrenergic alpha 1 Receptor"[tw] OR "Adrenergic alpha 1 Receptors"[tw] OR "Adrenergic alpha 1A Receptor"[tw] OR "Adrenergic alpha 1A Receptors"[tw] OR "Adrenergic alpha 1B Receptor"[tw] OR "Adrenergic alpha 1B Receptors"[tw] OR "Adrenergic alpha 1D Receptor"[tw] OR "Adrenergic alpha 1D Receptors"[tw] OR "alpha 1 Adrenergic Receptor"[tw] OR "alpha 1 Adrenergic Receptors"[tw] OR "alpha 1A Adrenergic Receptor"[tw] OR "alpha 1A Adrenergic Receptors"[tw] OR "alpha 1B Adrenergic Receptor"[tw] OR "alpha 1B Adrenergic Receptors"[tw] OR "alpha 1C Adrenergic Receptor"[tw] OR "alpha 1C Adrenergic Receptors"[tw] OR "alpha 1D Adrenergic Receptor"[tw] OR "alpha 1D Adrenergic Receptors"[tw] OR "alpha1 Adrenergic Receptor"[tw] OR "alpha1 Adrenergic Receptors"[tw] OR "alpha1A Adrenergic Receptor"[tw] OR "alpha1A Adrenergic Receptors"[tw] OR "alpha1B Adrenergic Receptor"[tw] OR "alpha1B Adrenergic Receptors"[tw] OR "alpha1C Adrenergic Receptor"[tw] OR "alpha1C Adrenergic Receptors"[tw] OR "alpha1D Adrenergic Receptor"[tw] OR "alpha1D Adrenergic Receptors"[tw] OR "alpha1 Adrenoreceptor"[tw] OR "alpha1 Adrenoreceptors"[tw] OR "alpha 1 Adrenoreceptor"[tw] OR "alpha 1 Adrenoreceptors"[tw] OR "alpha 1A Adrenoreceptor"[tw] OR "alpha 1A Adrenoreceptors"[tw] OR "alpha 1B Adrenoreceptor"[tw] OR "alpha 1B Adrenoreceptors"[tw] OR "alpha 1C Adrenoreceptor"[tw] OR "alpha 1C Adrenoreceptors"[tw] OR "alpha 1D Adrenoreceptor"[tw] OR "alpha 1D Adrenoreceptors"[tw] OR "alpha1 Adrenoreceptor"[tw] OR "alpha1 Adrenoreceptors"[tw] OR "alpha1A Adrenoreceptor"[tw] OR "alpha1A Adrenoreceptors"[tw] OR "alpha1B Adrenoreceptor"[tw] OR "alpha1B Adrenoreceptors"[tw] OR "alpha1C Adrenoreceptor"[tw] OR "alpha1C Adrenoreceptors"[tw] OR "alpha1D Adrenoreceptor"[tw] OR "alpha1D Adrenoreceptors"[tw] OR "alpha1 Adrenoceptor"[tw] OR "alpha1 Adrenoceptors"[tw] OR "alpha 1 Adrenoceptor"[tw] OR "alpha 1 Adrenoceptors"[tw] OR "alpha 1A Adrenoceptor"[tw] OR "alpha 1A Adrenoceptors"[tw] OR "alpha 1B Adrenoceptor"[tw] OR "alpha 1B Adrenoceptors"[tw] OR "alpha 1C Adrenoceptor"[tw] OR "alpha 1C Adrenoceptors"[tw] OR "alpha 1D Adrenoceptor"[tw] OR "alpha 1D Adrenoceptors"[tw] OR "alpha1 Adrenoceptor"[tw] OR "alpha1 Adrenoceptors"[tw] OR "alpha1A

Adrenoceptor"[tw] OR "alpha1A Adrenoceptors"[tw] OR "alpha1B Adrenoceptor"[tw] OR "alpha1B Adrenoceptors"[tw] OR "alpha1C Adrenoceptor"[tw] OR "alpha1C Adrenoceptors"[tw] OR "alpha1D Adrenoceptor"[tw] OR "alpha1D Adrenoceptors"[tw] OR (("alpha"[ti] OR alpha*[ti]) AND (adrenoreceptor*[ti] OR adrenoceptor*[ti] OR adrenergic receptor*[ti])) OR "peripheral sympathetic vasoconstriction"[tw])) OR (("smooth muscle"[ti] OR "Muscle, Smooth, Vascular"[majr]) AND ("vascular"[ti] OR "aortic"[ti] OR "vasoactive"[tw]) AND ("Receptors, Adrenergic, alpha 1"[majr] OR "Adrenergic alpha 1 Receptor"[ti] OR "Adrenergic alpha 1 Receptors"[ti] OR "Adrenergic alpha 1A Receptor"[ti] OR "Adrenergic alpha 1A Receptors"[ti] OR "Adrenergic alpha 1B Receptor"[ti] OR "Adrenergic alpha 1B Receptors"[ti] OR "Adrenergic alpha 1D Receptor"[ti] OR "Adrenergic alpha 1D Receptors"[ti] OR "alpha 1 Adrenergic Receptor"[ti] OR "alpha 1 Adrenergic Receptors"[ti] OR "alpha 1A Adrenergic Receptor"[ti] OR "alpha 1A Adrenergic Receptors"[ti] OR "alpha 1B Adrenergic Receptor"[ti] OR "alpha 1B Adrenergic Receptors"[ti] OR "alpha 1C Adrenergic Receptor"[ti] OR "alpha 1C Adrenergic Receptors"[ti] OR "alpha 1D Adrenergic Receptor"[ti] OR "alpha 1D Adrenergic Receptors"[ti] OR "alpha1 Adrenergic Receptor"[ti] OR "alpha1 Adrenergic Receptors"[ti] OR "alpha1A Adrenergic Receptor"[ti] OR "alpha1A Adrenergic Receptors"[ti] OR "alpha1B Adrenergic Receptor"[ti] OR "alpha1B Adrenergic Receptors"[ti] OR "alpha1C Adrenergic Receptor"[ti] OR "alpha1C Adrenergic Receptors"[ti] OR "alpha1D Adrenergic Receptor"[ti] OR "alpha1D Adrenergic Receptors"[ti] OR "alpha1 Adrenoreceptor"[ti] OR "alpha1 Adrenoreceptors"[ti] OR "alpha 1 Adrenoreceptor"[ti] OR "alpha 1 Adrenoreceptors"[ti] OR "alpha 1A Adrenoreceptor"[ti] OR "alpha 1A Adrenoreceptors"[ti] OR "alpha 1B Adrenoreceptor"[ti] OR "alpha 1B Adrenoreceptors"[ti] OR "alpha 1C Adrenoreceptor"[ti] OR "alpha 1C Adrenoreceptors"[ti] OR "alpha 1D Adrenoreceptor"[ti] OR "alpha 1D Adrenoreceptors"[ti] OR "alpha1 Adrenoreceptor"[ti] OR "alpha1 Adrenoreceptors"[ti] OR "alpha1A Adrenoreceptor"[ti] OR "alpha1A Adrenoreceptors"[ti] OR "alpha1B Adrenoreceptor"[ti] OR "alpha1B Adrenoreceptors"[ti] OR "alpha1C Adrenoreceptor"[ti] OR "alpha1C Adrenoreceptors"[ti] OR "alpha1D Adrenoreceptor"[ti] OR "alpha1D Adrenoreceptors"[ti] OR "alpha1 Adrenoceptor"[ti] OR "alpha1 Adrenoceptors"[ti] OR "alpha 1 Adrenoceptor"[ti] OR "alpha 1 Adrenoceptors"[ti] OR "alpha 1A Adrenoceptor"[ti] OR "alpha 1A Adrenoceptors"[ti] OR "alpha 1B Adrenoceptor"[ti] OR "alpha 1B Adrenoceptors"[ti] OR "alpha 1C Adrenoceptor"[ti] OR "alpha 1C Adrenoceptors"[ti] OR "alpha 1D Adrenoceptor"[ti] OR "alpha 1D Adrenoceptors"[ti] OR "alpha1 Adrenoceptor"[ti] OR "alpha1 Adrenoceptors"[ti] OR "alpha1A Adrenoceptor"[ti] OR "alpha1A Adrenoceptors"[ti] OR "alpha1B Adrenoceptor"[ti] OR "alpha1B Adrenoceptors"[ti] OR "alpha1C Adrenoceptor"[ti] OR "alpha1C Adrenoceptors"[ti] OR "alpha1D Adrenoceptor"[ti] OR "alpha1D Adrenoceptors"[ti] OR (("alpha"[ti] OR alpha*[ti]) AND (adrenoreceptor*[ti] OR adrenoceptor*[ti] OR adrenergic receptor*[ti])) OR "peripheral sympathetic vasoconstriction"[ti])) OR (("Heart Failure"[majr] OR "heart failure"[ti] OR "cardiac failure"[ti] OR "myocardial failure"[ti] OR "heart decompensation"[ti] OR "heart decompensation"[ti] OR "cardio-renal syndrome"[ti] OR "cardiorenal syndrome"[ti] OR "paroxysmal dyspnea"[ti] OR "cardiac edema"[ti] OR "cardiac oedema"[ti] OR "heart edema"[ti] OR "heart oedema"[ti] OR "failing myocardium"[ti] OR "failing heart"[ti] OR "failing human myocardium"[ti] OR "failing human heart"[ti]) AND (norepinephrine*[ti] OR "Norepinephrine/pharmacology"[majr])))
